# A Descriptive, Longitudinal Study of Quality of Life and Perceived Health Needs in Patients With Head and Neck Cancer

**DOI:** 10.6004/jadpro.2016.7.6.6

**Published:** 2016-09-01

**Authors:** S. Kate Sandstrom, Susan R. Mazanec, Haley Gittleman, Jill S. Barnholtz-Sloan, Nancy Tamburro, Barbara J. Daly

**Affiliations:** 1 Seidman Cancer Center, University Hospitals Case Medical Center, Cleveland, Ohio;; 2 Case Western Reserve University School of Medicine, Cleveland, Ohio

## Abstract

Patients with head and neck cancer have numerous concerns and symptoms in the first year of posttreatment survivorship and are especially vulnerable at the end of treatment and 1 month posttreatment. This article shares the findings of a descriptive, longitudinal study of health-related quality of life (HRQOL) in patients with head and neck cancer from the beginning of treatment through 12 months posttreatment. The primary objective of this study was to describe the symptom experience and health needs of patients receiving radiation for head and neck cancer to support the establishment of an advanced practitioner (AP) clinic for head and neck cancer survivors. Significant findings in this study showed HRQOL at the end of treatment was significantly lower than baseline (*p* < .001). Low scores persisted through 1 month, with gradual recovery by 12 months. Fatigue and anxiety had the highest mean scores, yet anxiety improved with time, whereas fatigue did not. Positive human papillomavirus status was statistically associated with higher anxiety. Socioeconomic status negatively impacted HRQOL. Themes of perceived health needs were managing oral symptoms, returning to a normal life, and regaining energy. The AP in oncology can play a pivotal role in providing comprehensive assessment, symptom management, health education, and supportive counseling in this population throughout treatment and survivorship.

It is estimated that by the year 2020, there will be close to 18 million cancer survivors in the United States, a 30% increase from 2010 (National Cancer Institute [NCI], 2014; Hebdon, Abrahamson, McComb, & Sands, 2014). Despite the recognition of the importance of survivorship care in oncology, there currently are no well-established guidelines for models of care or structured survivorship services (Lester, Wessels, & Jung, 2014; Howell et al., 2012). This is particularly true of care for patients afflicted with head and neck cancer (HNC).

Approximately 650,000 new cases of HNC are diagnosed each year worldwide. In the United States, approximately 61,760 will develop HNC in 2016, and HNC will account for 3% to 5% of all cancers (Siegel, Miller, & Jemal, 2016). Of particular note, the rate of oropharyngeal cancer (OPC) is 2 to 3 times the national rate in 7 neighborhoods in Cleveland, Ohio (the site of the current study), with 11 neighborhoods having double the rate of laryngeal cancer (Cuyahoga County Board of Health, 2013). Survival from HNC is one of the lowest among all malignancies, including breast, colon, and prostate cancers. The 5-year survival rate for all stages and types of HNC has not improved significantly in recent decades and remains approximately 63% overall (American Cancer Society [ACS], 2016).

Death rates for OPC have been decreasing over the past 3 decades, partly due to a decrease in smoking; however, there has been a global increase in the incidence of human papillomavirus (HPV)-related OPC, particularly in North America and Northern Europe (ACS, 2016; Gillison, Chaturvedi, Anderson, & Fakhry, 2015). In certain subsets of OPC, HPV, especially HPV-16, is now considered to be the leading cause of the increased incidence (Gillison et al., 2015). Despite this, HPV-related HNCs seem to have a better response to chemotherapy and radiation than do non–HPV-related HNC, with better overall and disease-free survival (Chaturvedi, 2012; Gillison, 2016).

Health-care reform and rising costs are at the forefront of political discourse, with more and more emphasis on preventive care and a healthy lifestyle. The total cost of cancer care from diagnosis to death is projected to be $174 billion by the year 2020 (NCI, 2014). Adding to the concern about the cost of care, workforce shortages are also expected, with significant shortages of oncology specialists and primary care physicians projected over the next decade (Dulko et al., 2013). With the increasing numbers of cancer survivors, other alternative care models are needed to meet the health needs of these cancer survivors (Ness et al., 2013).

The primary focus for oncologic follow-up care has typically been surveillance for recurrence and management of side effects. Over the past few decades, more emphasis has been placed on health-related quality of life (HRQOL), and its impact on survivorship has become an important component in cancer care. Often, after patients have completed curative treatments, follow-up care may be left to primary care providers, whose knowledge of the late effects and risks for development of other health problems may be inadequate (Taylor & Monterosso, 2015). However, the alternative plan for follow-up care with the oncologist may not be realistic or sustainable for the growing cancer survivor population.

Advanced practitioners (APs) in oncology with their specialty expertise and skills for decision-making as well as clinical acumen, are well suited to meet these challenges. Additionally, oncology nurses worldwide are already deeply engaged in survivorship care (Grant, Economou, & Ferrell, 2010). A systematic review by Howell et al. (2012) suggests that advanced practice nurse (APN)-led clinics and primary care follow-up may be equivalent in detecting recurrence when compared with more traditional oncologist-led care and that patient satisfaction is high with care delivered in APN-led clinics. Health institutions, however, often do not support the role of the AP in dedicated survivorship care, and this role is often poorly defined, with a wide range of variability in services (Lester et al., 2014). Moreover, we are aware of no established standards for HNC survivorship care, and few longitudinal studies are exploring the survivorship needs of this population.

## BACKGROUND

Head and neck cancer is strongly associated with environmental and lifestyle risk factors, including the use of tobacco products, alcohol consumption, certain occupational exposures, and a number of strains of the sexually transmitted HPVs. Patients with HNCs often present at a late stage, with many patients having poor physical and psychological health, poor social support, lower socioeconomic and education levels, and lack of medical care and exposure to healthier lifestyle practices (Harrison, Sessions, & Hong, 2009). Many patients also have other comorbid diseases such as chronic pulmonary disease, heart and vascular conditions, and dietary and vitamin deficiencies.

Treatment for HNC may have a significant impact on function and body image. Most patients with HNC receive combination therapies such as chemoradiotherapy, surgery, and radiation or a combination of all three modalities. Patients undergoing radiation treatment experience a rigorous daily radiation treatment regimen, often combined with chemotherapy, typically for 5 to 7 weeks depending on the stage and location of the cancer. Treatment breaks are avoided due to an increased risk for cancer regrowth and lowered chances for a cure. The severity of adverse effects may be based on the location and extent of the tumor as well as the type and extent of treatment delivery. Slow recovery rates and high morbidity with a wide range of late effects of treatment are common.

Survivors of HNCs have particularly unique and often debilitating consequences of cancer treatment, significantly impacting HRQOL and survivorship. A number of late effects of treatment are transient, whereas others may require a lifetime of management secondary to damage, resulting in more permanent dysfunction and disfigurement. Radiation-induced neural damage and pain may surface years after radiotherapy completion, sometimes making identification of the source of pain difficult (Chaplin & Morton, 1999). The risk of osteoradionecrosis developing after radiotherapy is highest during the first 3 to 24 months but persists throughout the patient’s life and may increase over time, with poor dentition and/or dental trauma (Eades, Chasen, & Bhargava, 2009). Furthermore, resultant deterioration in functional status, an inability to cope, distress, and chronic depression are also associated with poor outcomes (Shinn et al., 2016). Many late effects such as difficulty swallowing, chronic pain, xerostomia, and fatigue may persist and may have significant impact on QOL and survival.

A variety of QOL studies have been conducted over the past 3 decades in this population, revealing a complex and often confusing picture on the impact of late effects on survivorship (Murphy, Ridner, Wells, & Dietrich, 2007). A systematic review by Klein et al (2014) of HRQOL in patients being treated with radiation noted that effects from treatment significantly impacts HRQOL during the first 3 months, with a gradual return to near-normal baseline levels by 1 year post treatment. Several studies have described the impact of late physical treatment effects on overall survival and quality of life, as well as the wide variability and differences in recovery from treatment (Machtay et al., 2008; Langendijk et al., 2008; Yao et al., 2007). Yet there are few descriptive studies exploring how treatment influences patient perception of the impact of treatment burden and the long-term effects on overall health needs and survivorship.

Measurement of HRQOL has become an important assessment in oncology practice over the past decade, and such results may be important in predicting survival (Østhus, Aarstad, Olofsson, & Aarstad, 2013). A number of well-validated and tested HRQOL instruments are available for HNC; however, there is broad heterogeneity and varying obstacles to patients completing these instruments, especially when undergoing treatment for HNC. In a review by Ojo et al. (2012), a number of studies utilizing HRQOL instruments were evaluated and compared. The authors concluded that when addressing treatment alternatives, the patient’s HRQOL is a significant issue, and it is no longer enough to evaluate only mortality and morbidity. Howren et al. (2013) pointed out that HNC is the most psychologically traumatic cancer to experience and that information from HRQOL studies should not be ignored.

Survivors of HNC face numerous and complex challenges that require specialized care beyond the acute treatment period. It is the general recommendation that survivorship care should focus on concerns related to fear of recurrence, chronic fatigue, depression, employment and financial concerns, and late effects of cancer treatment (Stull, Snyder, & Demark-Wahnefried, 2007; Howell et al., 2012). Greater understanding of the unique concerns and needs of survivors of HNC is needed to expand typical models of survivorship care (Grant et al., 2010) to establish a format for delivery of the complex care needed by this patient population.

## STUDY AIMS

The purpose of this study was threefold. The first aim was to describe the symptom experience of patients with HNC receiving radiation therapy and the 12 months following completion of treatment in a large, urban academic cancer center. A second aim was to examine the clinical and demographic factors associated with levels of symptom distress, HRQOL, and health-related needs. The final aim was to evaluate patients’ perception of health-related needs during the 12-month recovery period. It was anticipated that these data would provide the foundation for developing an AP-led survivorship program for HNC survivors.

## METHODS

**Design, Setting, and Sample**

After attaining study approval by the institutional review board at the University Hospitals Case Medical Center, a convenience sample of 60 patients beginning initial radiation therapy for HNC was attained from the Department of Radiation Oncology at the University Hospitals Seidman Cancer Center, a NCI-designated comprehensive cancer center. A descriptive, correlational longitudinal design was used to examine symptom distress, HRQOL, and health-related needs in patients from the start of therapy through 12 months post completion of the treatment course.

Inclusion criteria were as follows: (1) age 18 and older; (2) diagnosis of cancer of the oral cavity, pharynx, larynx, salivary glands, or paranasal sinuses; (3) stages II, IIA, IIB, III, IVA, IVB disease; (4) undergoing initial primary curative treatment with radiation therapy or combined-modality therapy, including radiation therapy (i.e., combined radiation with chemotherapy; combined radiation with surgical resection; combined radiation, chemotherapy, and surgical resection); and (5) an Eastern Cooperative Oncology Group (ECOG) performance status of 0 to 2 at the time of enrollment. Exclusion criteria included: (1) prior radiation therapy for HNC; (2) stage IVC HNC due to a higher risk of recurrence and/or metastatic disease; and (3) patients who have legal guardians due to cognitive impairment or those who have a diagnosis of dementia.

**Measures**

*Demographic/Medical Information*: Demographic data were obtained from all participants at initial baseline enrollment. Additional medical information and staging were obtained from the participant’s medical record. Baseline comorbidity was assessed using the Charlson Comorbidity Index, which is a widely used and validated tool designed to assess the presence and type of 19 comorbid conditions and to predict the 10-year mortality rate for patients who have a range of comorbid conditions (Charlson, Pompei, Ales, & MacKenzie, 1987).

*Quality of Life*: The University of Washington Quality of Life Questionnaire (UW-QOL) version 4 is a questionnaire with 12 domain questions pertaining to symptoms, 3 questions related to HRQOL, 1 question asking patients to rank the 3 domains most important to them in the previous 7 days, and 1 question requiring patients to describe in writing any additional issues perceived as important to QOL. Respondents indicate the severity of each symptom within the past 7 days using hierarchical response options scaled from 100 (best) to 0 (worst). For example, to assess chewing, response options are "I can chew as well as ever" (100), "I can eat soft solids but cannot chew some foods" (50), and "I cannot even chew soft solids" (0). Swallowing is assessed with the response options of "I can swallow as well as ever" (100), "I cannot swallow certain solid foods" (67), "I can only swallow liquid food" (33), and "I cannot swallow because it ‘goes down the wrong way’ and chokes me" (Rogers & Lowe, 2012).

The composite score is the mean of the scores from the 12 symptom domains. This tool was developed to screen patients for dysfunction and to define criteria, so patients and problem domains can be identified by the clinical team, thereby making it possible to arrange for additional support and intervention if needed (Rogers & Lowe, 2009). Rogers and Lowe (2009) reported the test-retest reliability coefficient at 0.95.

*Psychological Distress*: The Brief Profile of Mood States Short Form–Revised (POMS-Brief) is a 30-item measure of psychological distress. Alternate forms and internal consistency indices of reliability of POMS-Brief indicate that the shorter forms possess comparable—and in some cases superior—reliability, relative to the original 65-item POMS, and is less time-consuming and taxing for ill patients with cancer to complete (McNair, Lorr, & Droppleman, 1992; Baker, Denniston, Zabor, Polland, & Dudley, 2002). It is a multiple-item assessment questionnaire that asks patients to describe their feelings during the past week.

The questions posed require answers ranging from 0 (not at all) to 5 (extremely). The POMS-Brief has six factor-based subscales in which data are summarized as scores on six mood states: tension, depression, anger, vigor, fatigue, and confusion. Scores are combined to yield a total mood disturbance score, with higher scores indicating greater mood disturbance. Reported alpha coefficients for the POMS-Brief range from 0.75 to 0.91 across all 6 subscales (McNair et al., 1992; Baker et al., 2002).

*Symptom Distress*: The Symptom Distress Thermometer is a self-administered scale developed by the National Comprehensive Cancer Network (NCCN, 2007; Vitek, Rosenzweig, & Stollings, 2007). The "distress thermometer" measures distress on a scale of 0 to 10, and a problem checklist identifies more specific etiologies of distress, such as practical, spiritual, physical, emotional, and family problems. The distress tool used in this study employed a Likert-type scale of 0 to 10 (0 = no distress; 10 = the most severe distress). A problem checklist was added to identify physical and psychosocial concerns for qualitative analysis, but it was not used in scoring the distress level.

*Qualitative Assessment*: At baseline and all follow-up time points, participants were asked an open-ended question: "What are your perceived health needs now and as you continue to recover?" Major themes were extracted from the data and summarized.

**Data-Collection Process**

Participants were screened for eligibility and provided informed consent prior to beginning radiation therapy by the radiation oncology APN. Study participants were followed for a 12-month period, with questionnaires administered at 6 time points: baseline (prior to beginning treatment); end of radiation treatment; and 1, 3, 6, and 12 months postradiation. Efforts were made to meet with patients face-to-face at these time points by the radiation oncology APN or the radiation social worker who was specifically trained by the APN to administer the questionnaires. If participants were unable to be in clinic at these times, follow-up phone calls were made by the APN in an attempt to complete the questionnaires over the phone. If this was not possible, forms were mailed to participants to complete and return.

**Data Analyses**

Frequency tables with means, standard deviations, and medians were generated to describe the distribution of the main outcomes (total UW-QOL, total POMS, and distress score) at each of the six time points. The POMS outcome was further broken down into subscales of tension-anxiety, depression, anger-hostility, vigor-activity, fatigue, and confusion-bewilderment. Paired t-tests were used to compare the means of each time point with the baseline measure for each of the main outcomes and POMS subscales. Spaghetti plots were drawn to see the trend over time of the main outcomes and POMS subscales for each study subject. For each of the main outcomes and POMS subscales, covariates including age, gender, marital status, insurance, socioeconomic status, race, tobacco use, HPV status, alcohol use, and drug use were assessed univariately and multivariately to see which ones should be included in longitudinal models. The final random-intercept longitudinal models for each of the main outcomes and POMS subscales included treatment, age, gender, insurance status, race, and HPV status.

**Sample**

A total of 118 patients were screened between August 2011 and February 2013, with 60 (51%) eligible patients consenting to participate in the study. The majority of the 60 patients (n = 44 or 73%) completed questionnaires at least 3 months post treatment, with 27 patients not completing the full 12 months of follow-up. Of the 27 nonresponders, 6 had progressive disease or died; 4 decided not to continue to participate after completion of treatment; 5 dropped out after the 1 month follow-up; 4 did not return forms or follow-up calls at the 6- and 12-month follow-up; 6 patients did not complete forms at 12 months; 1 patient missed his appointment at 1, 3, and 6 months; and 1 missed his appointment at 6 months.

Of the 60 enrolled patients, 59 received radiation treatment for HNC. One patient enrolled but did not complete treatment. The majority of patients had cancers of the oral cavity and pharynx (78.33%), and disease was stage III–IVA in 85%. A total of 48 patients (80%) were treated with concurrent chemotherapy and radiation (1 did not complete treatment), and the remaining patients were treated with radiation alone or surgery and radiation. See Table 1 for sample patient characteristics.

**Table 1:**
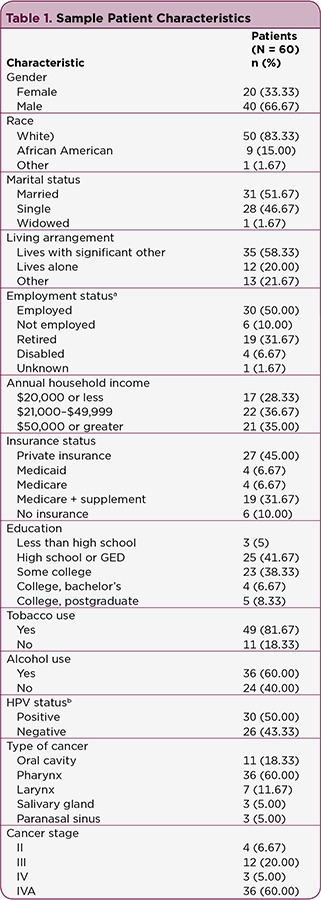
Sample Patient Characteristics

**Table 1:**
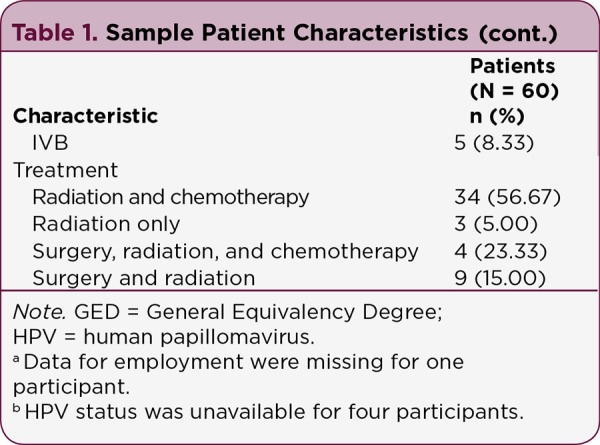
Sample Patient Characteristics (cont.)

## RESULTS

As shown in Table 2, mean scores for HRQOL, total mood disturbance, and distress showed similar trends throughout the 12-month period. Evaluation of UW-QOL scores indicated a significant decline in HRQOL at the completion of radiation therapy, which persisted through 1 month with gradual recovery by 12 months; however, there was variability in all 12 domains. The top 3 concerns from the UW-QOL (end of treatment through 12 months) were consistently saliva, taste, and swallowing. The total POMS score showed a similar trend, with low scores at the end of treatment and at 1 month. Again, scores improved by 12 months, with significant variability observed. Of the six POMS subscales, fatigue and anxiety had the highest mean scores; anxiety improved with time, but fatigue did not.

**Table 2:**
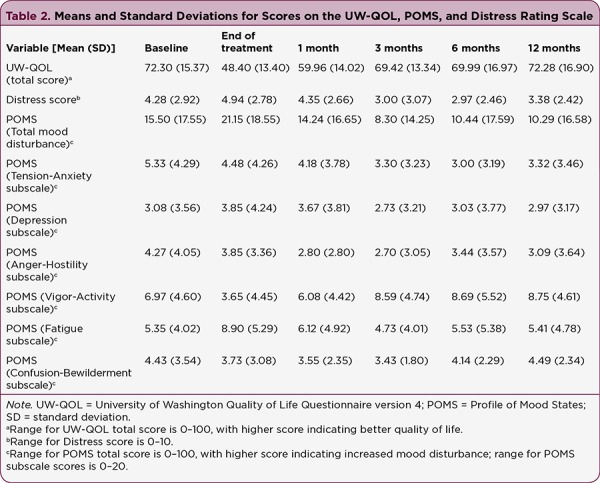
Means and Standard Deviations for Scores on the UW-QOL, POMS, and Distress Rating Scale

Depression scores were noted to be higher at baseline, at the end of treatment, and at 1 month, but they were not statistically significant. Distress scores also showed similar trends, although again they were not statistically significant. Patients did experience gradual recovery at 12 months, but spaghetti plots illustrated significant variability in recovery in those patients who completed the study (see Figure below).

Univariate longitudinal regression models identified statistically significant relationships between lower socioeconomic status and lower HRQOL (estimate = –7.81, *p* = .02) and between older age and lower scores for vigor/activity POMS subscale (estimate = –0.09, *p* = .03). However, these relationships did not hold in the multivariate models. The only statistically significant finding in the multivariate longitudinal regression models was that white race was associated with higher scores on the POMS confusion-bewilderment subscale (estimate = –1.89, *p* = .02).

Findings related to HPV status approached significance in the multivariate models. Positive HPV status was associated with higher scores on both the POMS confusion-bewilderment subscale (estimate = 1.13, *p* = .05) and the POMS tension-anxiety subscale (estimate = 1.81, *p* = .08). Additionally, the relationship between having no insurance and higher scores on the POMS tension-anxiety subscale approached significance (estimate = –2.72, *p* = .06) (see Table 3).

**Figure:**
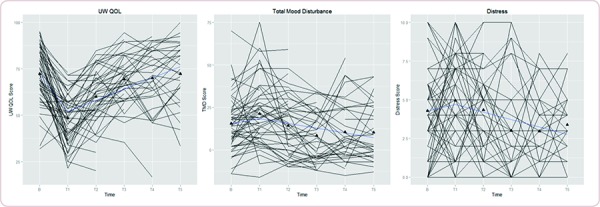
Spaghetti plots for UW-QOL, total mood disturbance, and distress. Blue lines indicate mean scores. UW-QOL = University of Washington Quality of Life Questionnaire version 4.

Perceived health-related needs were identified at all six time points and broken down into three major themes based on patient response: oral symptoms/needs, normalcy (socialization, finances, employment, staying healthy), and energy (fatigue, exercise, nutrition). Themes of perceived health needs were managing oral symptoms, returning to a normal life, and regaining energy. In addition, most patients expressed worry over recurrence and being "rid" of cancer. A number of patients also expressed a need for smoking cessation, more education on treatment, as well as recovery and staying healthy.

## DISCUSSION

This longitudinal study contributes to the literature by describing the symptom burden and impact on HRQOL in patients with HNC who are undergoing radiation therapy. There were six major findings in this study: (1) HRQOL scores at the end of radiation treatment were significantly lower than baseline; (2) low HRQOL scores persisted through 1 month, with gradual recovery by 12 months, although scores were highly variable; (3) of the POMS subscales, fatigue and anxiety had the highest mean scores; (4) positive HPV status was statistically associated with higher anxiety; (5) lower socioeconomic status negatively impacted HRQOL; and (6) the most frequent concerns were related to saliva, taste, swallowing, and pain.

Although the most frequent concerns were related to saliva, taste, swallowing, and pain, there was wide variability in symptoms across all time points. Oral symptoms and pain were more predominant at the end of treatment and at 1-month follow-up. Even though changes in HRQOL and symptom burden in our population mirror findings from other studies in the literature (Klein et al., 2014), a "one-size-fits-all" approach to patient management is unlikely to be effective in this vulnerable population. The immediate post-treatment transition clearly is a critical period for support, and moreover, it further illustrates the need for comprehensive assessment by an AP throughout this trajectory of time.

Consistent with findings by Klein et al. (2014), many participants also expressed frustration and disappointment with the slowness of recovery at 1 month and beyond. Many expected to have resolution of symptoms and to feel "normal" again. This certainly demonstrates the need to better prepare patients and caregivers on the trajectory of recovery, expected outcomes, and possible setbacks.

Even though our study did not show statistically significant differences in depression scores, major depressive disorders have been reported in up to 40% of patients with HNC, most commonly within the first 3 months of diagnosis (Chiou et al., 2013). Depression may be chronic, with resultant deterioration in functional status and inability to cope, and may contribute to poor outcomes. It is also a risk factor for malnutrition and other chronic effects and has been associated with lower overall survival and high morbidity (Loimu et al., 2015; Lydiatt, Denman, McNeilly, Puumula, & Burke, 2008). Because of the high risk for depression, it is essential for patients to be assessed throughout the course of treatment and survivorship.

Our study also underscores the need for awareness of vulnerable subgroups in this population. Positive HPV status was associated with higher anxiety; The reason for this is not clear but may possibly be related to younger age in the HPV-positive participants. Human papillomavirus–related oral cancers do not have the traditional risk factors of tobacco and alcohol exposure but rather are related to the sexually transmitted HPV virus (Lajer & Von Buchwald, 2010), which may have contributed to higher anxiety.

Lower socioeconomic status had a negative impact on HRQOL in this study, which was not surprising, as it has historically been linked to both higher incidence of and lower survival in patients with HNCs (Johnson, McDonald, & Corsten, 2008; Schleiffarth, Charlton, & Pagedar, 2013). Our institution draws from a very urban and inner city population as well as surrounding suburban communities. Only 50% of the patients were employed at baseline, 10% were unemployed, and the others were either retired or disabled.

**Study Limitations**

This study has several limitations, including a relatively small sample and high dropout rate over time, with a final number of 34 of 60 participants completing the entire 12 months. Overall, this is a challenging population to study due to the severity of side effects and grueling treatment regimens. We attempted to limit the amount of time required to complete the study forms and therefore may not have captured the full impact of treatment on participants’ HRQOL and symptom distress. Because depression has a high prevalence in this population and may have an impact on survival outcomes (Howren et al., 2013; Shinn, et al., 2016), we thought the POMS-Brief tool may not have adequately evaluated depression in this particular population. Despite these weaknesses, the results of this study underscore the critical need for involvement by APs in the development of survivorship services and care models.

## CLINICAL IMPLICATIONS

Despite advances in cancer treatment and survivorship, HNC continues to be linked to low survival rates, with significant physical and emotional sequelae impacting HRQOL. Patients with HNC have numerous concerns and symptoms in the first year of posttreatment survivorship and are especially vulnerable at the end of treatment and at 1 month posttreatment. The radiation oncology practitioner plays a pivotal role in providing comprehensive assessment, symptom management, health education, and supportive counseling. Advanced practitioners should be cognizant of not only the acute side effects of treatment but also the long-term and late effects of radiation in this patient population. Exercise programs and nutritional counseling should also be employed in the recovery process as part of rehabilitation and reintegration to a "new normal." Standardized screening tools, if not too burdensome, or brief questionnaires to evaluate physical and emotional distress, may guide the process and provide continuity of care (Rogers, 2016). Referrals to specialists such as speech therapists, physical and occupational therapists, and psychiatric and counseling services prior to treatment and long term should also be considered as part of survivorship standard practice.

Routine survivorship follow-up is essential in oncologic care, with ongoing assessment and data collection throughout the recovery process. Survivorship visits and treatment summaries should optimally be done within the first 3 months post treatment, with the goal by 1 month. With current projections of cancer survivors over the next decade, planning and coordinating health-care needs are imperative. Instituting these measures would ensure the standardization of care, with the goal of improved survival outcomes and HRQOL.

At our institution, we have now developed a preliminary survivorship program in radiation oncology for our HNC patients to have a scheduled visit with the radiation oncology APN at 1 month post treatment. A comprehensive treatment summary and follow-up plan are reviewed with the patient and caregiver along with a complete physical exam. Health promotion with 12-month goals is also established with the patient.

Finally, with the enormous economic burden of oncologic care and often deleterious impact on employment, patients and families may need financial and employment counsel (Liu, 2008). Financial concerns were identified by patients throughout the 12-month trajectory of the study. Appropriate referral to financial counselors and agencies could be facilitated with the assistance of social work and community outreach programs and organizations.

**Table 3:**
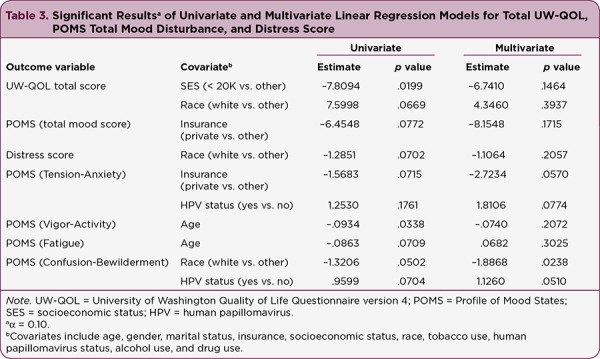
Significant Results of Univariate and Multivariate Linear Regression Models for Total UW-QOL, POMS Total Mood Disturbance, and Distress Score

Although this is a small study, the impact of treatment in this patient population is apparent. Moreover, the inclusion of oncology APs in the management of these patients would be of great benefit. Future research is needed to evaluate the cost-effectiveness and utilization of an AP-run survivorship clinic as a model of care for this challenging population. Intervention studies are also needed to test approaches to improving posttreatment function. With the surge in the number of APs over the past several years, oncology APs are now poised to be agents of change in the survivorship arena and meet the growing demands and care needs of the public.

**Acknowledgment**

The authors would like to offer special thanks to Susan R. Mazanec, PhD, RN, AOCN®, for her guidance and mentorship in this project.
